# Imagined and extended contact experiences and adolescent bystanders' behavioral intentions in homophobic bullying episodes

**DOI:** 10.1002/ab.22059

**Published:** 2022-11-04

**Authors:** Raquel António, Rita Guerra, Lindsey Cameron, Carla Moleiro

**Affiliations:** ^1^ Centro de Investigação e Intervenção Social Iscte ‐ Instituto Universitário de Lisboa Lisboa Portugal; ^2^ Applied Psychology Research Center Capabilities and Inclusion (APPsyCI) Ispa‐ Instituto Universitário Lisbon Portugal; ^3^ School of Psychology University of Kent Canterbury UK

**Keywords:** bystanders, extended contact, homophobic bullying, imagined contact

## Abstract

Bystanders' helping interventions in bias‐based bullying are rare, although they have the potential to intervene on behalf of the victim and quickly stop the aggression. Two studies tested, experimentally, the impact of adolescents' imagined (Study 1, *N* = 113, *M*_age_ = 16.17) and extended contact experiences (Study 2, *N* = 174, *M*_age_ = 15.79) on assertive bystanders' behavioral intentions in the context of homophobic bullying, an under‐researched but highly detrimental behavior that emerges mainly during early adolescence. Potential mediators (empathic concern, social contagion concerns, and masculinity/femininity threat) were also examined. Results showed that female younger participants revealed more behavioral intentions to help victims of homophobic bullying when asked to imagine an interaction with an outgroup member (Study 1). Younger participants revealed less masculinity/femininity threat in the positive extended contact condition, and female participants revealed less empathic concern in the negative extended contact condition (Study 2). Overall, these findings identify specific conditions (e.g., younger females) where indirect contact interventions (i.e., extended and imagined) are likely to have a stronger impact. Age and sex differences were found to illustrate how adolescents vary in their behavioral intentions, empathic concern, and threat; and also highlight the need to further examine age and sex differences regarding responses to homophobic bullying episodes.

## 1 INTRODUCTION

Research on bullying has traditionally focused on the victims and aggressors, taking an individualistic approach to the phenomenon. However, several recent studies consider bullying as a group phenomenon, highlighting the importance of the role of peers, given that they are present in more than 80% of bullying episodes (e.g., Jungert et al., 2020; Lynn Hawkins et al., 2001). These peers, usually known as bystanders, can endorse different roles such as encouraging the aggressor or helping the victim (Polanin et al., 2012; Salmivalli et al., 1996). Bystanders' behaviors in aggressive victimization, such as bias‐based bullying episodes (i.e., bullying towards a socially marginalized group) are considered to be a key factor in stopping child and adolescent peer victimization (e.g., Palmer & Abbott, 2017). However, assertive behavioral intervention by bystanders (i.e., to help the victim or stop the perpetrator) is rare (e.g., Frey et al., 2015).

To further understand the decision‐making process young bystanders engage in when deciding when and how to intervene, a branch of developmental psychological research has been focusing on identifying predictors of bystanders' assertive behavioral *intentions* (i.e., their intentions to help the victims). Bystander intentions are behaviors young people report they would engage in when confronted with an instance of bullying or victimization (Abbott & Cameron, 2014; Mulvey et al., 2016; Palmer et al., 2015). This is often measured by presenting young people with realistic but hypothetical bullying or victimization scenarios, together with a number of potential bystander response options, which are rated. This research has focused on identifying different individual, interpersonal, group, and intergroup factors that may influence how children/adolescents understand and evaluate bullying acts. Individual‐level factors, such as high levels of self‐efficacy, openness, and empathy have been related to bystander‐defending behavioral intentions (e.g., Abbott & Cameron, 2014; Salmivalli, 2014) and may interact with group processes to predict bystanders' behavioral intentions toward bullying (e.g., ingroup identification and intergroup contact; Palmer & Abbott, 2017).

The present research focuses on assertive bystander behavioral intentions (i.e., to help, or defending), and extends previous studies by investigating bystanders' behavioral intentions in *homophobic bullying* situations, an under‐researched, but a highly detrimental form of bias‐based bullying (Espelage et al., 2008). Sexual minorities are at greater risk of bullying and cyberbullying (Llorent et al., 2016) and this aggressive behavior emerges mainly during early adolescence (Espelage, Basile, et al., 2018; Toomey & Russell, 2016), when homophobic name‐calling is common (Espelage, Valido, et al., 2018). However, literature reviews and interventions rarely focus on homophobic school‐based victimization (Toomey & Russell, 2016).

Identifying means of increasing bystanders' assertive behavioral intentions in homophobic bullying incidents is important for efforts to tackle this form of bullying. With this in mind, we propose that there may exist specific predictors, and underlying mechanisms, that inhibit or facilitate bystanders' assertive behavioral intentions in homophobic bullying episodes. In two studies, we aim to: (a) test, experimentally, the impact of two indirect contact interventions on adolescents' bystander behavioral intentions to a homophobic bullying incident; (b) examine intergroup factors that are specific to the homophobic bullying context (i.e., masculinity and femininity threat and social contagion concerns), while also (c) considering the developmental period (i.e., between middle adolescence and late adolescence) in which these prosocial behaviors occur, and (d) considering sex differences in its effects.

### 1.1 Intergroup contact and bystander behavioral intentions in homophobic bullying

Extensive research has established the importance of one of the most influential theories of prejudice reduction: the intergroup contact theory. The “contact hypothesis” (Allport, 1954) posits that intergroup contact is an efficient strategy to improve intergroup relations and reduce prejudice in different contexts and age groups (e.g., see Pettigrew & Tropp, 2006 for meta‐analysis; Turner, Hewstone, et al., 2007; Vezzali et al., 2017). Importantly, there are specific contexts where there is little to no opportunity for direct contact, such as in segregated contexts, or when the outgroup identity is invisible (e.g., sexual minorities or people with a mental illness; White et al., 2020). Consequently, recent research has focused on the effectiveness of indirect forms of contact (e.g., extended contact, imagined contact, and vicarious contact), where contact is not face‐to‐face. More than replacing direct contact or overcoming its limitations, exploring indirect forms of contact promotes an understanding of underlying psychological processes that may encourage future direct contact experiences (White et al., 2020).

Indirect forms of contact have also been linked to positive intergroup relations in childhood and adolescence, in a number of intergroup contexts (e.g., affecting attitudes towards migrants, Stathi et al., 2014; lesbian women and gay men, Turner, West, et al., 2013; refugees; Vezzali et al., 2015; see Turner & Cameron, 2016, for a review).

Imagined intergroup contact is a form of indirect contact that consists of mentally simulating a positive interaction with a member or members of an outgroup using participants' imagination (Crisp & Turner, 2009). Imagining an interaction with an outgroup member may be used in less diverse or segregated contexts and may prepare people for future direct contact (Miles & Crisp, 2014; Stathi et al., 2014). Meta‐analytic research has shown that imagined contact is effective at reducing prejudice towards a variety of social groups and in different contexts (e.g., elderly, ethnic, national, and religious outgroups; see Miles & Crisp, 2014, for review). A recent intervention with elementary school children showed that imagined contact increased intentions to counteract social exclusion and bullying of disabled children, as well as helping intentions and willingness for outgroup contact (Vezzali, Birtel, et al., 2019). Specific to the current intergroup context, imagined intergroup contact has been shown to reduce prejudice towards gay men (Turner, Crisp, et al., 2007).

Besides imagined contact, indirect contact can also take the form of extended contact, which involves knowing a member of one's own group who is friends with a member of another group (Wright et al., 1997). Meta‐analytic evidence from 20 years of research on extended contact has shown its effectiveness in improving intergroup relations (Zhou et al., 2018). Research with adults revealed the positive impact of extended contact with lesbian women and gay men on homophobic behaviors and attitudes, via reduced anxiety and reduced sexual prejudice (Mereish & Poteat, 2014). Extended contact has also been shown to increase behavioral intentions to meet gay/lesbian people, via more perceived moral purity (Vezzali et al., 2017). Recent studies with adolescents have also shown that those with friends with gay or lesbian friends (i.e., extended contact) showed more assertive bystanders' behavioral intentions to help homophobic bullying victims (António et al., 2017). This is particularly important, given that research shows that intentions strongly predict actual behaviors (e.g., Smith & McSweeney, 2007).

Indeed, research suggests that contact interventions may be also useful in bullying, for instance, in improving helping responses to bias‐based bullying situations (Palmer & Abbott, 2017). No research to date has examined the impact of imagined contact on adolescents' bystander behavioral intentions in homophobic bullying. Based on existing findings and extending them to bystanders' behavioral intentions towards victims of homophobic bullying, we aim to examine, experimentally, in two studies, the effect of imagined and extended contact on adolescents' behavioral intentions to help victims of homophobic bullying.

Extensive research and theory on indirect contact have examined the positive effect of indirect contact on attitude and behavior change through different social processes (White et al., 2020). Research has shown different underlying mechanisms through which intergroup contact positively impacts intergroup relations (e.g., greater empathy, less intergroup anxiety, and less threat; Davies et al., 2011; Pettigrew & Tropp, 2008; Turner, Hewstone, et al., 2007). Pettigrew and Tropp's (2008) review on how intergroup contact reduces prejudice focused on the three most‐studied mediators of contact effects (i.e., increased knowledge of the outgroup, increased empathy, and decreased intergroup anxiety). Importantly, in this specific context, other potential mediators might be important to identify and to better understand why indirect contact experiences have positive consequences for intergroup relations. Thus, besides examining the effects of the abovementioned intergroup factors on bystanders' behavioral intentions, we will also examine one common mediator of contact effects (i.e., empathic concern) and explore two underlying mechanisms specific to this context (i.e., social contagion concerns and masculinity/femininity threat) that may account for their impact on bystanders' behavioral intentions to help homophobic bullying victims.

### 1.2 Empathic responses, social contagion, and masculinity/femininity threat

#### 1.2.1 Empathic responses

Research consistently shows that empathy is related to more helping and prosocial behaviors and lower prejudice (Abbott & Cameron, 2014; Gini et al., 2007; Katz et al., 2014). Some authors proposed that empathy encompasses four empathy states, two referring to perspective taking (i.e., imagine‐self perspective and imagine‐other perspective) and two others to emotional responses (i.e., emotion matching and empathic concern; Batson & Ahmad, 2009). Empathic concern is an *other‐oriented* emotion evoked by perceiving someone in need and involves feelings of sympathy, compassion, and tenderness for those who are in need (Batson & Ahmad, 2009). According to Batson et al. (2007), the empathic concern is an affective form of empathy that can motivate helping behaviors and is related to more positive attitudes and helping intentions towards outgroup members and stigmatized groups (e.g., homeless individuals and people with AIDS; e.g., Batson et al., 1997). Specifically related to bystanders' helping behavioral intentions, research showed that undergraduates reported more empathic concern and more intentions to help a victim of party rape when the potential victim was a friend, rather than a stranger (Katz et al., 2014). Indeed, empathy is one of the underlying mechanisms through which intergroup contact improves intergroup behaviors (e.g., Dovidio et al., 2010) and bystander intentions (Abbott & Cameron, 2014). In their work with adolescents, António et al. (2017) found that, besides decreasing masculinity/femininity threat, extended contact also increased affective empathy towards the victims of homophobic bullying and, through that, increased assertive bystanders' behavioral intentions (António et al., 2017). This is consistent with research showing that other forms of indirect contact, such as imagined contact, also lead to more empathy and less prejudice (Kuchenbrandt et al., 2013). Based on these findings, our research focuses on the relationship between indirect contact, “empathic concern,” and bystander intentions. We propose that, in the context of homophobic bullying, indirect contact experiences will increase feelings of sympathy, compassion, and tenderness for the victims (i.e., empathic concern), leading in turn to more bystander' behavioral intentions to help bullying victims.

#### 1.2.2 Social contagion

“Social contagion” refers to the phenomenon whereby individuals are concerned that contact with stigmatized group members (e.g., lesbian and gay people) results in misclassification as an outgroup member (Buck, 2010; Buck et al., 2013). These concerns regarding misclassification as gay or lesbian have been associated with several negative outcomes. Research shows that concerns over being misidentified as gay or lesbian are related to negative attitudes towards sexual minorities (e.g., Cascio & Plant, 2016), denigration, and avoidance of lesbian and gay people (Plant et al., 2014). It is feasible that social contagion concerns present an additional deterrent to helping victims of homophobic bullying, as bystanders fear stigma and “contagion” and run the risk of becoming themselves the target (Pichardo, 2015). However, most studies to date focused on the intergroup consequences of these concerns in adults, and less are known about its impact among youth, with some exceptions described below. Buck et al. (2013) examined whether social contagion concerns have implications for intergroup contact with lesbian and gay individuals, beyond levels of sexual prejudice. Studies conducted with college students revealed that social contagion concerns independently predict anxiety and avoidance in response to imagined, anticipated, and actual contact with a lesbian or gay individual, after controlling for negative attitudes toward homosexuality (Buck et al., 2013). Consistent with previous findings with adults, social contagion concerns among adolescents were negatively related to their intentions to help a victim of homophobic bullying (António et al., 2018). This negative effect occurred via more negative attitudes towards lesbians and gay men in general. These findings provided preliminary evidence for the negative consequences of social contagion concerns in the context of bystanders' behavioral intentions in homophobic bullying episodes. Importantly for the current studies, research has shown that positive imagined contact (i.e., imagining having contact with a famous gay or lesbian person) reduces concerns of misidentification as gay or lesbian (i.e., contagion concerns; Lacosse & Plant, 2018).

This type of concern may be particularly important for adolescents due to the pressure they usually experience to behave according to traditional gender norms by society, parents, and peers (Espelage, Valido, et al., 2018). Deviating from these norms may result in victimization, often in the form of homophobic bullying (Espelage, Valido, et al., 2018). Therefore, students who do not behave according to traditional gender roles, like traditional masculinity, are more likely to be harassed based on their actual or perceived sexual orientation or gender identity (Espelage, Valido, et al., 2018). In this sense, bystanders who help a victim of homophobic bullying may also become the object of abuse or misclassification as gay or lesbian by associating with the victim. The fear of being misidentified by associating with a victim of homophobic bullying may influence adolescents' decision of helping.

Given the effectiveness of the imagined contact paradigm against harsh forms of discrimination and high‐prejudiced contexts (White et al., 2020), we aim to examine the effect of imagined contact on bystanders' behavioral intentions to help victims of homophobic bullying, by reducing these social contagion concerns. No research to date has examined the influence of extended contact on contagion concerns, but there is reason to believe that extended contact may be effective for reducing contagion concerns as it is at reducing prejudice (e.g., Zhou et al., 2018) and threats to masculinity and femininity (António et al., 2017). Therefore, in these studies, we propose that indirect contact (i.e., imagined and extended) will increase adolescents' behavioral intentions to help the victims of homophobic bullying by reducing these social contagion concerns.

#### 1.2.3 Masculinity/femininity threat

Intergroup contact theory suggests that contact positive effects on intergroup relations occurs through changes in both cognitive and emotional factors (e.g., increased knowledge, increased empathy, and decreased threat). Indeed, meta‐analytical research has shown that perceived threat is a key mediator of contact effects to reduce prejudice and improve intergroup attitudes (e.g., Pettigrew & Tropp, 2008). The present research examines a form of threat specific to this intergroup context, masculinity/femininity threat, that is directly related to the domain of bullying based on gender and sexual orientation. Masculinity/femininity threat appears when manhood (or womanhood) is questioned and is usually related to antigay attitudes and negative behaviors towards those who threatened this identity (e.g., Reese et al., 2013; Talley & Bettencourt, 2008). Indeed, homosexuality may be considered a threat to masculinity (Falomir‐Pichastor & Mugny, 2009). Boys aim to demonstrate masculinity from a young age, to avoid being bullied and labeled gay (Phoenix et al., 2003). Importantly, given the concealable nature of sexual orientation, heterosexual privilege is threatened simply by associating with sexual minorities (Duhigg et al., 2010). Moreover, inducing a threat to masculinity/manhood (i.e., a public gender role violation) increases motivation to engage in aggressive behaviors (Bosson et al., 2009). Physical aggression following these threats diminishes anxiety caused by the threat (Bosson et al., 2009). Thus, we argue that feelings of threat to masculinity and femininity may prevent youth from engaging in assertive behavioral intentions when witnessing homophobic bullying incidents.

It is essential to explore strategies that may reduce masculinity/femininity threats and thereby increase bystander assertive behavioral intentions. Previous research with adolescents has tested the extended contact paradigm in the context of masculinity/femininity threat, and bystander intentions. António et al. (2017) found that heterosexual adolescents who reported having heterosexual friends who have gay/lesbian friends (i.e., extended contact) reported more assertive bystanders' behavioral intentions, and this was partially accounted for by decreased masculinity/femininity threat. We aim to extend this research and experimentally examine its effects on bystanders' behavioral intentions to help victims of homophobic bullying, by reducing masculinity/femininity threat. No research to date has examined the influence of imagined contact on this type of threat. However, previous research has shown that imagined contact is effective at reducing prejudice and stereotype threat (e.g., Vezzali et al., 2013), and contagion concerns (Lacosse & Plant, 2018) in adults. Therefore, there is reason to expect that imagined contact may be effective for reducing masculinity/femininity threat as it is at reducing prejudice and stereotype threat (e.g., Vezzali et al., 2013), and contagion concerns (Lacosse & Plant, 2018). We propose that indirect contact (i.e., imagined and extended) will increase adolescents' behavioral intentions to help the victims of homophobic bullying by reducing masculinity/femininity threats.

In sum, the current research examines, for the first time, empathic concern, social contagion, and masculinity/femininity threat in relation to adolescent bystanders' behavioral intentions toward victims of homophobic bullying, and the impact of indirect contact interventions on these variables.

## 2 EXPERIMENT 1

The main goal of Experiment 1 is to examine the effect of imagined contact on bystanders' assertive behavioral intentions, among adolescents (15–19 years) in homophobic bullying incidents. Overall, we predicted that participants in the imagined contact condition will reveal more assertive behavioral intentions, more empathic concern, less social contagion concerns, and less masculinity/femininity threat, compared with participants in the control condition (H1). Previous research (Abbott & Cameron, 2014) found that young people aged 11–13 years were more likely to report they would intervene in a group‐based bullying incident if they had experienced more intergroup contact. On the basis of research, but considering the lack of previous research examining age differences on the tested variables across adolescence, age was included in the analysis for exploratory reasons, with no specific hypotheses being formulated regarding its moderator role. Testing the impact of imagined contact on bystander behavioral intentions, also allowed us to determine the effectiveness of this technique and make age‐specific recommendations for future school‐based interventions using these methods (Cameron & Rutland, 2016).

Homophobic attitudes and behaviors are usually associated with masculinity norms and beliefs (e.g., Poteat & Vecho, 2016), and research consistently show that male adolescents have more negative attitudes toward sexual minorities (e.g., Costa & Davies, 2012), higher levels of aggression and bullying than females (Pepler et al., 2006); and female adolescents score higher in defending behaviors in bullying episodes than male adolescents (e.g., Pozzoli & Gini, 2012). Girls also generally report higher empathic responses than boys (e.g., Caravita et al., 2009). Based on these findings, we further expected that female participants (vs. male), in the imagined contact condition, would show more assertive behavioral intentions, more empathic concern, less social contagion concerns, and less femininity threat (H1a).

Finally, we predicted that the effect of imagined contact on assertive behavioral intentions would be mediated by increased empathic concern, and also decreased social contagion concerns, and decreased threat to masculinity/femininity (H1b).

## 3 METHODS

### 3.1 Participants and procedure

Participants were 124 students (78 female) from one public and one private Portuguese school, aged between 15 and 19 years (*M* = 16.19, SD = 1.11), enrolled in 10th (51%) and in 12th grade (35%). The majority of participants identified as heterosexual (91%) and the remaining as gay/lesbian or bisexual, did not respond or declared having doubts as to their sexual orientation. Since we investigated bullying towards gay/lesbian adolescents, the final sample included only participants who identified as heterosexual (113 students; *M*age = 16.17, SD = 1.09; 73 female). Participants were divided into two groups according to their age/development period: middle adolescence (<16 years) and late adolescence (>16 years).

The survey was approved by the institutional Ethics Committee and conducted in accordance with the ethical standards of the American Psychological Association, the Declaration of Helsinki, and the European General Data Protection Regulation. Additionally to their own agreement to participate, informed consent information was provided. Participants completed a paper and pencil questionnaire in classrooms with a teacher and the researcher. Participants were randomly assigned to either an imagined contact condition or a control condition, based on Turner, Crisp, et al. (2007). Participants in the imagined contact condition were asked to imagine a conversation with a gay boy/lesbian girl [sex matched to participant] who sat next to them on the train and to list the interesting and unexpected things they discovered about him/her following the imagined conversation. Participants assigned to the control condition were asked to imagine they were on a 3‐day hiking trip and to list the different things they saw in the imagined scene (see supporting information for full imagined contact instructions). Finally, participants filled out a sex‐matched questionnaire with the measures of interest. The first part of the questionnaire was composed of 8 questions designed to gather demographic data and complete the experimental manipulation, followed by a distraction task. The second part of the questionnaire, including the dependent measures and mediators, was presented as a different study. After completing the questionnaires, all students received a written debriefing.

### 3.2 Measures^1^, ^2^

#### 3.2.1 Empathic concern

Participants were presented with a name‐calling homophobic bullying vignette where the victim matched the participant's sex. Empathic concern towards the victim was measured with four items (e.g., “*I feel sympathy for the bullied boy*”), on a 7‐point scale (1 = not at all to 7 = very much; *α* = .86), adapted from Katz et al. (2014). Higher scores indicate more empathic concern.

#### 3.2.2 Social contagion concerns

On the basis of the previous research (e.g., Buck et al., 2013) to measure social contagion concerns, participants were asked to indicate, on a 7‐point scale (1 = strongly disagree to 7 = strongly agree), to what extent they agreed or disagreed with eight statements related to contagion concerns (e.g., “*If I was hanging out with a gay/lesbian person, I would worry that other people would think I was gay/lesbian, too*”). Following Buck et al. (2013), we created a composite score of social contagion (*α* = .89), where higher values indicate higher social contagion concerns.

#### 3.2.3 Masculinity/femininity threat

We adapted Reese et al. (2013) measure of masculinity/femininity threat. Participants were asked to what extent they agreed or disagreed with three statements (e.g., “*I would feel my masculinity/femininity threatened if a gay boy/lesbian girl flirted with me*”) on a 7‐point scale (1 = strongly disagree to 7 = strongly agree). We created a composite score of threat, where higher values indicate higher perceived threat (*α* = .78).

#### 3.2.4 Assertive behavioral intentions

Based on previous studies (Abbott & Cameron, 2014; Palmer & Cameron, 2010; Palmer et al., 2015) to measure bystanders' behavioral intentions, participants read a vignette of a name‐calling homophobic bullying episode (matching participant's sex) and indicated their intention to engage in 10 different bystander behavioral intentions. In this study, we focused only on assertive bystander behavioral intentions (four items, on a 5‐point scale, 1 = never do to 5 = always do; e.g., *“I would try and make student B feel better,”* *α* = .70).

## 4 RESULTS

### 4.1 Imagined contact effects

First, we conducted 2 experimental condition (imagined contact vs. control) × 2 sex (female vs. male) × 2 age group (middle adolescence vs. late adolescence) multivariate analysis of variance (MANOVA) to examine the impact of the manipulation and predicted moderators on our main dependent variables. Moderated mediation analysis was used to test the conditional indirect effect of the experimental condition on assertive bystanders' behavioral intentions, through empathic concern, social contagion concerns, and masculinity/femininity threat. Contrary to the expected, the multivariate effect of the experimental condition was not significant, Wilks' λ = 0.989, *F*(4, 101) = 0.28, *p* = .89, *η* ^2^_*p*_ = 0.011. Also, the two‐way interactions between the experimental condition and age, and experimental condition and sex were nonsignificant (*p* > .05). Even so, the main effect of sex was significant, Wilks' *λ* = 0.699, *F*(4, 101) = 10.85, *p* < .001, *η* ^2^_*p*_ = 0.301, and the main effect of age group was also significant, Wilks' *λ* = 0.893, *F*(4, 101) = 3.04, *p* = .02, *η* ^2^_*p*_ = 0.107. Significant univariate effects were found for some of the dependent variables, as described below.

#### 4.1.1 Assertive bystanders' behavioral intentions

The univariate results revealed a significant three‐way interaction between the experimental condition, age group, and sex, *F*(1, 104) = 4.82, *p* = .030, *η* ^2^_*p*_ = 0.044. Simple contrasts comparing imagined contact versus control conditions showed that female younger participants revealed more assertive behavioral intentions in the imagined contact condition, relative to the control condition (see Table 1). Additionally, results revealed a significant main effect of sex, *F*(1, 104) = 17.09, *p* < .001, *η* ^2^_*p*_ = 0.141, such that female participants showed more assertive bystanders' behavioral intentions (*M* = 3.94, SD = 0.65) than male participants (*M* = 3.30, SD = 0.75). Overall, partially confirming H1a and H1b, younger female participants showed more assertive behavioral intentions in the imagined contact condition.

**Table 1:** Means, standard deviations, main effects, and interaction effects by condition (*N* = 112) (Experiment 1)

	Sex		Age group		Younger adolescents		Older adolescents	
	F	M	Younger	Older	F	M	F	M
*Assertive behavioral intentions*								
Imagined contact	3.99 (0.72)	3.41 (0.87)	4.06 (0.72)	3.60 (0.84)	4.31 (0.62)^a^	3.46 (0.60)	3.75 (0.72)	3.39 (0.99)
Control	3.90 (0.58)	3.19 (0.61)	3.71 (0.63)	3.61 (0.71)	3.75 (0.66)^b^	3.55 (0.54)	4.04 (0.46)	3.07 (0.60)
*Social contagion concerns*								
Imagined contact	2.13 (1.20)	3.18 (1.19)	2.65 (1.38)	2.42 (1.25)	2.22 (1.21)	3.73 (1.26)	2.06 (1.22)	2.94 (1.13)
Control	2.48 (1.32)	2.94 (1.54)	3.03 (1.59)	2.38 (1.22)	2.84 (1.53)	3.73 (1.76)	2.15 (1.00)	2.68 (1.43)
*Masculinity/femininity threat*								
Imagined contact	2.56 (1.58)	3.95 (1.78)	3.21 (1.89)	2.98 (1.72)	2.47 (1.57)	5.06 (1.31)	2.63 (1.63)	3.48 (1.78)
Control	2.66 (1.42)	3.95 (1.71)	3.33 (1.63)	2.96 (1.65)	2.91 (1.45)	4.87 (1.39)	2.42 (1.38)	3.64 (1.74)
*Empathic concern*								
Imagined contact	5.54 (1.17)	4.45 (1.56)	5.48 (1.34)	4.94 (1.44)	5.92 (1.04)	4.38 (1.44)	5.26 (1.20)	4.48 (1.66)
Control	5.45 (1.23)	4.19 (1.52)	5.27 (1.29)	4.83 (1.55)	5.24 (1.24)	5.40 (1.61)	5.66 (1.21)	3.78 (1.30)

*Note*: Means with different subscripts in each column indicate differences at *p* < .050.

Abbreviations: F, female; M, male.

The main effect of the experimental condition, the interaction between the experimental condition and age, and the interaction between the experimental condition and sex were not significant.

#### 4.1.2 Mediators: Empathic concern, social contagion concerns, and masculinity/femininity threat

*Empathic concern*: The results revealed a significant three‐way interaction between the experimental condition, age group, and sex, *F*(1, 104) = 6.38, *p* = .01, *η* ^2^_*p*_ = 0.158. We conducted simple contrasts comparisons, however these were not significant. Additionally, results revealed a significant main effect of sex, *F*(1, 104) = 13.23, *p* < .001, *η* ^2^ *_p_* = 0.113. Female participants showed more empathic concern (*M* = 5.50, SD = 1.19) than male participants (*M* = 4.32, SD = 1.52).

The univariate results showed that the main effect of the experimental condition, the interaction between the experimental condition and age, and the interaction between the experimental condition and sex were not significant.

*Social contagion concerns*: Results revealed a significant main effect of sex, *F*(1, 104) = 12.11, *p* = .001, *η* ^2^_*p*_ = 0.104, and age, *F*(1, 104) = 5.97, *p* = .016, *η* ^2^_*p*_ = 0.054. Female participants showed less social contagion concerns (*M* = 2.31, SD = 1.26) than male participants (*M* = 3.06, SD = 1.37). Also, social contagion concerns were greater among younger (*M* = 2.85, SD = 1.49) than among older participants (*M* = 2.40, SD = 1.22).

The univariate results showed that the main effect of the experimental condition and the interaction effects with age group and sex were not significant.

*Masculinity/femininity threat*: Results revealed a significant main effect of sex, *F*(1, 104) = 24.41, *p* < .001, *η* ^2^_*p*_ = 0.190, and age, *F*(1, 104) = 5.44, *p* = .022, *η* ^2^_*p*_ = 0.050. Female participants showed less masculinity/femininity threat (*M* = 2.61, SD = 1.49) than male participants (*M* = 3.95, SD = 1.73). Also, the masculinity/femininity threat was greater among younger (*M* = 3.27, SD = 1.74) than among older participants (*M* = 2.97, SD = 1.67).

The univariate results showed that the main effect of the experimental condition and the interaction effects with age group and sex were not significant.

### 4.2 Indirect effects of imagined contact

Next, we tested the conditional indirect effect of the experimental condition on assertive bystanders' behavioral intentions, through empathic concern, social contagion concerns, and masculinity/femininity threat with PROCESS bootstrapping macro (Model 8; Hayes, 2013) for SPSS with 5000 resamples and 95% percentile bootstrap confidence interval (CI). The experimental condition was the predictor; age group and sex were the moderators; empathic concern, social contagion, and threat were the mediators; and assertive bystanders' behavioral intentions were the outcome. The experimental condition was dummy‐coded (control = 0; imagined contact = 1). Contrary to hypothesized (H1c), none of the indexes of moderated mediation was significant (see Table 2 and Supporting Information).

**Table 2:** Imagined contact's indirect effect on assertive behavioral intentions (Experiment 1)

	*M* (social contagion)			*M* (threat)			*M* (empathic concern)			*Y* (assertive bystanders)		
	Coefficient	SE	*p*	Coefficient	SE	*p*	Coefficient	SE	*p*	Coefficient	SE	*p*
Constant	1.39**	0.37	.00	3.98**	0.52	.00	6.61**	0.38	.00	2.92**	0.36	.00
(*X*) Dummy	−.13	0.24	.58	−.05	0.30	.87	.16	0.25	.53	.10	0.11	.38
*M* (social contagion)	–	–	–	–	–	–	–	–	–	‐.01	0.05	.88
*M* (threat)	–	–	–	–	–	–	–	–	–	‐.01	0.04	.93
*M* (empathic concern)	–	–	–	–	–	–	–	–	–	.25**	0.05	.00
(*W*) Age	−.60*	0.25	.02	−.56	0.31	.08	−.29	0.26	.27	−.10	0.12	.43
*X* × *W*	.51	0.50	.31	.29	0.61	.64	−.20	0.51	.69	−.37	0.23	.12
	*R* ^2^ = 0.130			*R* ^2^ = 0.171			*R* ^2^ = 0.171			*R* ^2^ = 0.398		
	*F*(4, 107) = 3.992, *p* = .005			*F*(4, 107) = 5.514, *p* < .001			*F*(4, 107) = 5.508, *p* = .001			*F*(6, 105) = 11.555, *p* < .001		
Constant	3.51**	0.42	.00	3.17**	0.66	.00	3.51**	0.42	.00	2.65**	0.39	.00
(*X*) Dummy	−.13	0.24	.58	−.10	0.31	.75	−.13	0.24	.58	.10	0.12	.39
(*W* ^1^) Sex	.86**	0.26	.00	1.44**	0.32	.00	.86**	0.26	.00	‐.32*	0.13	.02
*X* × *W* ^1^	.53	0.50	.29	.04	0.62	.95	.53	0.50	.29	.09	0.24	.71
	*R* ^2^ = 0.130			*R* ^2^ = 0.169			*R* ^2^ = 0.170			*R* ^2^ = 0.384		
	*F*(4, 107) = 4.007, *p* = .005			*F*(4, 107) = 5.448, *p* = .001			*F*(4, 107) = 5.482, *p* = .001			*F*(6, 105) = 10.914, *p* < .001		

*Note*: The values are unstandardized regression coefficients.

* *p* < .05

** *p* < .01.

## 5 DISCUSSION

Findings from Experiment 1 partially supported the hypothesis that imagined contact with a gay/lesbian individual results in more positive outcomes compared to imagining something unrelated. Specifically, the results showed that younger female participants revealed more behavioral intentions to help victims of homophobic bullying when asked to imagine an interaction with an outgroup member. These findings are consistent with previous work, showing that imagined contact is related to more positive intergroup attitudes (e.g., Turner et al., 2007), and that there is a developmental decline in helping behaviors (e.g., Evans & Smokowski, 2015; Palmer & Abbott, 2017). However, the findings also highlight potential limitations of the imagined contact approach: Imagined contact was shown to be ineffective with older adolescents, and males, having an impact on bystander intentions *only* among younger females. Female students also revealed more empathic concern towards the victims, regardless of the experimental condition, and this is consistent with previous research showing that girls usually report higher levels of empathy than boys (e.g., Gini et al., 2007; Jenkins & Nickerson, 2017). Interestingly, the results showed the imagined contact effects are moderated simultaneously by sex and age, and not independently as we hypothesized. That is, contact was not more effective for female adolescents in general, but for the younger ones in particular. At the same time, and contrary to our hypotheses, imagined contact did not influence social contagion concerns and masculinity/femininity threats. The theoretical and practical implications of these results are further discussed in Section 10.

## 6 EXPERIMENT 2

The main goal of Experiment 2 was to examine the effect of extended contact interventions on bystanders' assertive behavioral intentions. Two forms of extended contact were tested, varying in valence, positive extended contact, and negative extended contact. Intergroup contact experiences can be positive and negative (e.g., Dixon & McKeown, 2021). Although research on positive *extended* contact is more prevalent than negative extended contact, previous research showed that negative events more strongly affect attitudes and that both positive and negative extended contact impacts intergroup relations (e.g., Mazziotta et al., 2015; Wölfer et al., 2017). A recently revised definition of extended contact poses that this kind of indirect contact consists of simply knowing about interactions between ingroup and outgroup members and can vary in degree of closeness and valence (Vezzali & Stathi, 2021). So, one can know about one's in‐group friends having positive or negative relationships with the out‐group (Wang et al., 2019). For instance, research shows that both positive and negative extended contact predicted intergroup attitudes, via their associations with positive and negative direct contact among German individuals (Mazziotta et al., 2015). Thus, not only positive but also negative extended contact might affect bystanders' attitudes toward the outgroup. In line with these findings, we will further consider the impact of negative extended contact on bystanders' helping behavioral intentions. Overall, we predicted that participants in a positive extended contact condition would reveal more assertive behavioral intentions, more empathic concern, less social contagion concerns, and less masculinity/femininity threat (H2).

As in Experiment 1, no specific hypotheses were formulated regarding age differences for the tested variables, but age was included in the analysis for exploratory reasons. Based on previous research (e.g., Costa & Davies, 2012; Evans & Smokowski, 2015), we further expected that positive extended contact would be more effective for female (vs. male) participants (H2a). Finally, we expected the positive effect of extended contact on bystanders' behavioral intentions to be mediated by more empathic concern, less social contagion concerns, and a reduced threat to masculinity/femininity (H2b).

## 7 METHODS

### 7.1 Participants and procedure

A total of 206 students (101 female) participated in the study, from two public Portuguese schools aged between 14 and 19 years (*M* = 15.81, SD = 1.22). Most participants (76%) were in high school (10th–12th years) and 24% were in middle school. Most participants identified as heterosexual (85%) and the remaining as gay/lesbian or bisexual did not respond or declared having doubts as to their sexual orientation. As in Experiment 1, the final sample included only participants who identified as heterosexual (174 students; *M*age = 15.79, SD = 1.23; 78 female). As in Experiment 1, participants were divided into two groups according to their age and development period: middle adolescence (<16 years) and late adolescence (>16 years).

The survey was approved by the institutional Ethics Committee and conducted in accordance with the ethical standards of the American Psychological Association, the Declaration of Helsinki, and the European General Data Protection Regulation. Data were collected in two public schools and additionally to their own agreement to participate after informed consent information was provided. Participants completed a paper and pencil questionnaire in classrooms with a teacher and the researcher. Based on previous research (Eller et al., 2015), we manipulated the valence of the extended contact (positive contact vs. negative contact vs. control) through fabricated entries on an Internet forum, in which an ingroup member (i.e., heterosexual) described his/her positive, negative or absence of contact with a member of the outgroup (i.e., gay/lesbian person). All participants were presented with an excerpt that started with an entry posted by a supposed lesbian or gay student who was moving to the school and asked the online community about the school environment since she/he had problems in her/his current school related to her/his sexual orientation. After that, all participants read one of three different replies to this message provided by a heterosexual student from their school, depending on the valence of the extended contact condition and participants' sex (see Supporting Information for full instructions).

Finally, after reading the messages, participants responded to the same measures used in Experiment 1 to assess bystanders' assertive behavioral intentions (*α* = .67), social contagion concerns (*α* = .87), masculinity/femininity threat (*α* = .78), and empathic concern (*α* = .83). The first part of the sex‐matched questionnaire was composed of eight questions designed to gather demographic data and complete the experimental manipulation, followed by a distraction task. The second part of the questionnaire, including the dependent measures and mediators, was presented as a different study. After completing the questionnaires, a written debriefing was delivered to each student.

## 8 RESULTS

### 8.1 Extended contact effects

First, we conducted a 3 experimental condition (positive extended contact vs. negative extended contact vs. control) × 2 sex (female vs. male) × 2 age group (middle adolescence vs. late adolescence) MANOVA to examine the impact of the manipulation and predicted moderators on our main dependent variables, since most of these variables were significantly related. Then, we conducted a moderated mediation to test the conditional indirect effect of the experimental condition on assertive bystanders' behavioral intentions, through empathic concern, social contagion concerns, and masculinity/femininity threat. Contrary to the expected, the multivariate effect of the experimental condition was not significant, Wilks' *λ* = 0.968, *F*(8, 308) = 0.64, *p* = .75, *η* ^2^_*p*_ = 0.016. The two‐way interactions between the experimental condition and age and the experimental condition and sex were also nonsignificant. However, the main effect of sex was significant, Wilks' *λ* = 0.732, *F*(4, 154) = 14.09, *p* < .001, *η* ^2^_*p*_ = 0.268, as well as the main effect of age group Wilks' *λ* = 0.931, *F*(4, 154) = 2.87, *p* = .03, *η* ^2^_*p*_ = 0.069. Significant univariate effects were found for some of the dependent variables, as described below.

#### 8.1.1 Assertive bystanders' behavioral intentions

Results revealed significant main effects of sex, *F*(1, 157) = 18.88, *p* < .001, *η* ^2^_*p*_ = 0.107 and age, *F*(1, 157) = 6.82, *p* = .010, *η* ^2^_*p*_ = 0.042. Female participants showed more assertive behavioral intentions (*M* = 3.98, SD = 0.69) than male participants (*M* = 3.43, SD = 0.87). Moreover, younger participants revealed more assertive bystanders' behavioral intentions (*M* = 3.84, SD = 0.76) than older participants (*M* = 3.52, SD = 0.89).

The univariate results showed that the main effect of the experimental condition, as well as the interaction effects with age group and sex, were not significant.

#### 8.1.2 Mediators: Empathic concern, social contagion concerns, and masculinity/femininity threat

*Empathic concern*: As predicted (H2a), results revealed an interaction between the experimental condition and sex, *F*(2, 157) = 2.76, *p* = .066, *η* ^2^_*p*_ = 0.034, although only approaching significance. Simple contrasts comparing positive contact versus negative contact, negative contact versus control and positive contact versus control conditions separately for female and male participants showed that female participants revealed less empathic concern in the negative contact condition, compared to the control condition (see Table 3). Additionally, results showed significant main effects of sex, *F*(1, 157) = 33.19, *p* < .001, *η* ^2^_*p*_ = 0.175, and age, *F*(1, 157) = 6.08, *p* = .015, *η* ^2^_*p*_ = 0.037. Regardless of condition, female participants showed more empathic concern (*M* = 5.63, SD = 1.16) than male participants (*M* = 4.34, SD = 1.55). Also, a main effect of the age group showed that empathic concern was greater among younger (*M* = 5.21, SD = 1.41) than among older participants (*M* = 4.63, SD = 1.57). Overall, the direct effect of extended contact was driven by negative contact triggering less empathic concern for female participants, partially supporting H2a.

**Table 3:** Means, standard deviations, main effects, and interaction effects by condition (*N* = 169) (Experiment 2)

	Sex		Age group		Younger adolescents		Older adolescents	
	F	M	Younger	Older	F	M	F	M
*Assertive behavioral intentions*								
Positive	4.04 (0.73)	3.38 (0.97)	3.98 (0.78)	3.45 (0.97)	4.11 (0.83)	3.81 (0.71)	3.96 (0.60)	3.08 (1.03)
Negative	3.88 (0.52)	3.39 (0.93)	3.69 (0.82)	3.52 (0.78)	3.95 (0.57)	3.44 (0.93)	3.78 (0.42)	3.34 (0.93)
Control	4.02 (0.83)	3.50 (0.77)	3.91 (0.67)	3.56 (0.90)	4.30 (0.39)	3.65 (0.70)	3.81 (1.02)	3.35 (0.77)
*Social contagion concerns*								
Positive	2.19 (0.83)	3.09 (1.54)	2.47 (1.22)	2.80 (1.40)	1.92 (0.68)	3.20 (1.42)	2.51 (0.92)	3.01 (1.65)
Negative	1.91 (0.76)	3.53 (1.81)	2.83 (1.66)	2.74 (1.63)	1.89 (0.68)	3.71 (1.85)	1.94 (0.92)	3.32 (1.80)
Control	2.13 (0.95)	3.03 (1.51)	2.81 (1.45)	2.48 (1.29)	2.10 (0.67)	3.28 (1.64)	2.15 (1.15)	2.77 (1.38)
*Masculinity/femininity threat*								
Positive	2.38 (1.17)	3.42 (1.87)	2.55 (1.40)^a^	3.24 (1.91)	2.02 (1.07)	3.25 (1.53)	2.82 (1.60)	3.54 (2.10)
Negative	2.35 (1.36)	4.32 (1.91)	3.68 (1.84)^b^	3.07 (2.06)	2.64 (1.42)	4.67 (1.66)	1.90 (1.18)	3.90 (2.18)
Control	2.75 (1.42)	3.77 (1.80)	3.68 (1.74)^b^	3.01 (1.60)	3.47 (1.63)	3.82 (1.84)	2.21 (0.98)	3.71 (1.74)
*Empathic concern*								
Positive	5.66 (1.07)^a,b^	4.35 (1.55)	5.50 (0.99)	4.54 (1.71)	5.78 (0.88)	5.13 (1.05)	5.52 (1.30)	3.83 (1.64)
Negative	5.29 (1.23)^a^	4.56 (1.28)	5.02 (1.43)	4.72 (1.09)	5.55 (1.39)	4.53 (1.33)	4.88 (0.85)	4.61 (1.25)
Control	5.98 (1.12)^b^	4.11 (1.83)	5.14 (1.76)	4.72 (1.86)	6.13 (1.00)	4.48 (1.88)	5.87 (1.23)	3.73 (1.76)

*Note*: Means with different subscripts in each column indicate differences at *p* < .050.

Abbreviations: F, female; M, male.

The univariate results revealed that the main effect of the experimental condition was not significant.

*Social contagion concerns*: Results revealed a significant main effect of sex, *F*(1, 157) = 29.07, *p* < .001, *η* ^2^_*p*_ = 0.156, such that female participants showed less social contagion concerns (*M* = 2.08, SD = 0.85) than male participants (*M* = 3.23, SD = 1.62).

The univariate results for the social contagion concerns showed that the main effect of the experimental condition, as well as the interaction effects with age group and sex, were not significant.

*Masculinity/femininity threat*: Results revealed a marginal interaction between the experimental condition and age group, *F*(2, 157) = 2.77, *p* = .066, *η* ^2^_*p*_ = 0.034. Simple contrasts comparing positive contact versus negative contact, positive contact versus control, and negative contact versus control showed that younger participants revealed less masculinity/femininity threat in the positive contact condition, relative to the control condition (see Table 3). Simple contrasts also showed that younger participants revealed lower masculinity/femininity threat in the positive contact condition, relative to the negative contact condition (see Table 3). In addition, results revealed a significant main effect of sex, *F*(1, 157) = 25.96, *p* < .001, *η* ^2^_*p*_ = 0.142, such that female participants showed less threat (*M* = 2.48, SD = 1.38) than male participants (*M* = 3.84, SD = 1.87). Overall, the direct effect of extended contact was driven by positive contact triggering less masculinity/femininity threat for younger participants.

The univariate results showed that the main effect of the experimental condition was not significant.

### 8.2 Indirect effects of extended contact

We tested the conditional indirect effect of the experimental condition on assertive bystanders' behavioral intentions, through empathic concern, social contagion concerns, and masculinity/femininity threat, with PROCESS bootstrapping macro (Model 8; Hayes, 2013) for SPSS with 5000 resamples and 95% percentile bootstrap CI. Experimental condition was the predictor, age group and sex were the moderators; empathic concern, social contagion, and threat were the mediators; and assertive bystanders' behavioral intentions were the outcome. The experimental condition was dummy‐coded (*d*_negative_: control = 0; positive = 0; negative = 1; and *d*_positive_: control = 0; positive = 1; and negative = 0). Contrary to the hypothesis (H2b), none of the indexes of moderated mediation were significant (see Table 4 and Supporting Information).

**Table 4:** Extended contact's indirect effect on assertive behavioral intentions (Experiment 2)

	*M* (social contagion)			*M* (threat)			*M* (empathic concern)			*Y* (assertive bystanders)		
	Coefficient	SE	*p*	Coefficient	SE	*p*	Coefficient	SE	*p*	Coefficient	SE	*p*
Constant	.89*	0.35	.01	1.15*	0.43	.01	6.89**	0.36	.00	4.64**	0.21	.00
(*X*) *D*_positive_	.06	0.25	.81	−.38	0.31	.22	.02	0.26	.94	−.07	0.15	.62
(cov) *D*_negative_	.16	0.26	.54	.06	0.31	.86	−.09	0.26	.73	−.14	0.15	.34
*M* (social contagion)	–	–	–	–	–	–	–	–	–	−.21**	0.05	.00
*M* (threat)	–	–	–	–	–	–	–	–	–	−.07*	0.04	.05
*M* (empathic concern)	–	–	–	–	–	–	–	–	–	.29**	0.04	.00
(*W*) age	−.08	0.21	.71	−.25	0.25	.33	−.50*	0.21	.02	−.34*	0.12	.01
*X* × *W*	.37	0.43	.40	1.13*	0.53	.03	−.42	0.45	.35	−.12	0.26	.64
	*R* ^2^ = 0.164			*R* ^2^ = 0.185			*R* ^2^ = 0.209			*R* ^2^ = 0.171		
	*F*(5, 163) = 6.413, *p* < .001			*F*(5, 163) = 7.398, *p* < .001			*F*(5, 163) = 8.597, *p* < .001			*F*(6, 162) = 5.565, *p* < .001		
Constant	2.73**	0.34	.00	3.52**	0.43	.00	5.72**	0.36	.00	3.00**	0.30	.00
(*X*) *D*_positive_	.07	0.25	.78	−.34	0.31	.28	.00	0.26	.99	−.05	0.12	.70
(cov) *D*_negative_	.18	0.26	.49	.11	0.32	.74	−.11	0.27	.79	−.10	0.12	.43
(*W* ^1^) Sex	1.15**	0.21	.00	1.36**	0.26	.00	−1.26**	0.21	.00	−.03	0.12	.82
*X* × *W* ^1^	−.36	0.43	.40	−.43	0.54	.42	.04	0.45	.94	−.18	0.21	.40
	*R* ^2^ = 0.164			*R* ^2^ = 0.166			*R* ^2^ = 0.205			*R* ^2^ = 0.429		
	*F*(5, 163) = 6.410, *p* < .001			*F*(5, 163) = 6.473, *p* < .001			*F*(5, 163) = 8.378, *p* < .001			*F* (7, 161) = 17.248, *p* < .001		
Constant	.90*	0.35	.01	1.22*	0.43	.01	6.89**	0.36	.00	2.77**	0.31	.00
(*X*) *D*_negative_	.17	0.26	.51	.08	0.32	.81	−.09	0.26	.73	−.10	0.13	.44
(cov) *D*_positive_	.07	0.25	.77	−.34	0.31	.28	.00	0.26	.99	−.05	0.12	.71
(*W*) Age	−.08	0.21	.71	−.25	0.26	.33	−.50*	0.21	.02	−.19	0.10	.06
*X* × *W*	−.12	0.44	.78	−.68	0.54	.21	.42	0.45	.35	.15	0.21	.48
	*R* ^2^ = 0.161			*R* ^2^ = 0.170			*R* ^2^ = 0.209			*R* ^2^ = 0.428		
	*F*(5, 163) = 6.261, *p* < .001			*F*(5, 163) = 6.697, *p* < .001			*F*(5, 163) = 8.597, *p* < .001			*F*(7, 161) = 17.198, *p* < .001		
Constant	2.79**	0.33	.00	3.71**	0.42	.00	5.69**	0.35	.00	2.98**	0.30	.00
(*X*) *D*_negative_	.16	0.25	.52	.09	0.32	.78	−.12	0.26	.65	−.10	0.13	.42
(cov) *D*_positive_	.06	0.25	.81	−.36	0.31	.25	−.01	0.26	.96	−.05	0.12	.71
(*W* ^1^) Sex	1.15**	0.21	.00	1.36**	0.25	.00	−1.26**	0.21	.00	−.03	0.12	.81
*X* × *W* ^1^	.73	0.43	.10	.95	0.54	.08	.86	0.45	.06	−.02	0.22	.91
	*R* ^2^ = 0.175			*R* ^2^ = 0.178			*R* ^2^ = 0.222			*R* ^2^ = 0.426		
	*F*(5, 163) = 6.911, *p* < .001			*F*(5, 163) = 7.055, *p* < .001			*F*(5, 163) = 9.302, *p* < .001			*F*(7, 161) = 17.074, *p* < .001		

*Note*: The values are unstandardized regression coefficients.

* *p* < .05

** *p* < .01.

## 9 DISCUSSION

Overall, partially supporting our predictions (H2a), we found an interaction between extended contact and sex on the empathic concern. In particular, female participants showed less empathic concern when reading about negative extended contact (H2a). Additionally, the effect of the valence of extended contact on masculinity/femininity threat was also moderated by age. Specifically, younger participants revealed lower masculinity/femininity threat in the positive contact condition, compared to negative extended contact and control. These findings are in line with prior work showing that extended contact is related to less masculinity/femininity threat and more empathy toward homophobic bullying victims (António et al., 2017). However, contrary to our hypotheses, there were no main effects of the experimental condition. At the same time, and contrary to our hypotheses, extended contact did not influence bystanders' behavioral intentions and social contagion concerns. These results are further discussed in Section 10.

## 10 GENERAL DISCUSSION

The aim of the current studies was to extend knowledge of bystanders' motivations to defend victims, specifically by examining the impact and limits of imagined and extended contact on bystanders' intentions and related variables in a very prevalent but understudied intergroup context of aggression‐homophobic bullying. We also explored developmental trends in key variables and the impact of the interventions among young and old adolescents and its effects among female and male adolescents. Taken together, the two experiments showed that: (a) imagining having contact with a lesbian or gay person promotes more bystanders' assertive behavioral intentions than imagining something unrelated, but this effect is limited to female younger participants (14–16 years) and (b) reading about a positive extended friendship triggered less threat compared to both negative extended contact and no contact experiences, but only among younger participants; while negative extended contact triggered less empathic concern, only among female participants.

Female younger participants revealed more behavioral intentions to help victims of homophobic bullying when asked to imagine a positive interaction with an outgroup member (i.e., a gay or lesbian person). Younger participants (both female and male) revealed lower masculinity/femininity threats in the positive contact condition, compared to negative extended contact and control. Additionally, female participants showed less empathic concern when reading about negative extended contact. Taken together, the findings build on existing research showing that extended contact is associated with lower levels of prejudice and stereotypes (e.g., Tam et al., 2009; Turner et al., 2013) while negative extended contact is related to less positive intergroup attitudes (e.g., Mazziotta et al., 2015). Additionally, these results are consistent with previous findings revealing that imagined contact is associated with increased helping intentions (Vezzali, Birtel, et al., 2019). The findings extend previous research by applying this approach to adolescents' bystander behavioral intentions during incidents of aggression (i.e., homophobic bullying), and identifying specific conditions (female, young age group) that the interventions are likely to have a more positive impact. For older adolescents and males, more rigorous interventions may be necessary to shift more entrenched and polarized views. Thus, the use of imagined contact interventions may need to be tailored to the age group to ensure its effectiveness, while maybe needing more intensive intervention for older participants and males. Also, imagining interacting with an outgroup member may be less consistent when it comes to changing behaviors, particularly high‐risk behaviors like defending homophobic bullying victims (e.g., Poteat & Vecho, 2016).

These results also complement and extend prior research by examining important intergroup processes involved in the developmental decline in bystander intentions, specifically, empathic concern, masculinity, and femininity threat, and social contagion concerns. Although the results did not reveal the expected indirect effects via these variables, we illustrated, for the first time, developmental trends in these variables across adolescence: regardless of the experimental manipulation, younger adolescents had higher behavioral intentions to help the victims and higher empathic concern. These findings are consistent with previous research, showing that younger adolescents are more likely to intervene as prosocial bystanders, compared to older ones (e.g., Evans & Smokowski, 2015).

In Experiment 1, contrary to our predictions, imagined contact was not effective in reducing masculinity and femininity threats and social contagion concerns. In Experiment 2, contrary to the expected, extended contact did not influence bystanders' behavioral intentions and social contagion concerns. These findings are also inconsistent with previous studies demonstrating the efficacy of direct intergroup contact in reducing intergroup threat (see Pettigrew & Tropp, 2008). The research has a number of theoretical implications. In Experiment 1, imagined contact did not affect the threat to masculinity/femininity, highlighting the potential limits of the imagined contact model. This lack of impact of imagined contact may be due to the lack of personal connection required in the imagined contact framework: unlike the direct intergroup contact experiences which involve personal relationships with the outgroup. It is also possible that the lack of significant effects of extended contact may be related to the operationalization of this variable. In the current research extended contact was manipulated through simple written instructions about an unknown person's (i.e., ingroup member) extended friendships and not about someone the participant actually knew, as in self‐reported measures of extended contact. We can speculate that this is a less personal way of triggering extended contact and future studies could test a more personal manipulation involving, for instance, the participants reflecting on actually extended friendships or test a more protracted intervention with repeated exposure to extended contact. Moreover, given that the experience of reading may be different from observing, future studies could test different forms of contact (e.g., vicarious contact), using manipulations that include the observation of positive intergroup contact, and which have been shown to be effective within educational settings (e.g., watching videos focusing on intergroup friendships between heterosexual and gay/lesbian individuals; Vezzali, Di Bernardo, et al., 2019). This may be more effective to increase assertive behavioral intentions, particularly among older boys. The findings also point to the importance of studying the effect of indirect contact (imagined and extended) on behavioral intentions and behaviors, within a wider social context. Youth are pressured by a majority‐status‐dominated society to behave according to traditional sex roles and to demonstrate behaviors like traditional masculinity, often to fit in with friends (Espelage, Valido, et al., 2018). It is possible that indirect contact interventions, removed from real‐world contexts may not be adequate to change behavioral intentions.

From very early youth learn to use insults often related to sexual orientation, sometimes not knowing their true meaning, but knowing that they must use them and avoid being the target of these insults to conform to the majority group's stereotypic expectations and norms. Youth are aware that sexual orientation is not readily identifiable and is hard to “prove,” so any heterosexual person can be inaccurately classified as LGB, which may result in concerns, and a threat, given the risk of experiencing prejudice and discrimination by being associated with this stigmatized group (Buck, 2010). As indicated by Lacosse and Plant (2018), traditional measures of indirect contact may not be as effective for reducing these concerns and threats to identity as it is to reduce prejudice, and future studies should include additional details (e.g., imagining contact with positive outgroup exemplars) or include different levels of personal involvement (e.g., knowing about ingroup members' outgroup friends ‐extended contact‐ from a social network; Vezzali & Stathi, 2021) to activate different processes and effectively reduce these concerns and threats to identity. Different forms of indirect contact that bridge the gap between imagined interactions, and real‐life behaviors (Abbott et al., 2019), may be required to reduce masculinity and femininity threats and social contagion concerns.

Further contributing to the existing knowledge of bystanders' intervention, and specifically the moderating impact of age and sex, our studies showed a developmental decline in bystander responses, with bystander assertive behavioral intentions being greater among younger adolescents than older adolescents. This is consistent with previous research showing a developmental decline in bystanders' helping behaviors and behavioral intentions across different age groups (e.g., Menesini et al., 1997; Palmer et al., 2015; Pepler & Craig, 1995; Rigby & Johnson, 2006). These developmental trends are consistent with the expectations that as youth age, they may feel increasingly afraid of intervening in incidents where not directly involved (Evans & Smokowski, 2015) and that it may be more socially acceptable to defend victims at younger ages (Ma et al., 2019).

This developmental variation may also be explained by different contextual information (e.g., group norms, group identity) alongside changes in social cognition or reasoning. Specifically, when making decisions on whether to help when witnessing bullying episodes, children, and adolescents need to balance moral and social information (Palmer et al., 2021). Recent research argues that developmental differences in helping behaviors depend on the context (who is being bullied), with older adolescents being more likely to help in some contexts, depending on the moral and social information they power to make the decision (e.g., in ethnic‐context—stigmatized victim vs. school context—nonstigmatized victim; Palmer et al., 2022, 2021). While intervention programs usually focus on younger youth (i.e., elementary and middle school; Mulvey et al., 2016), we believe that future intervention research should develop programs that encourage adolescents of all ages, especially older ones, to intervene and support bullying victims. This is particularly relevant for interventions that aim to tackle homophobic bullying among adolescents. Specifically, future school‐based interventions could consider adolescents' age and explore its effects over time with longitudinal data.

Importantly, and in line with previous research on defending attitudes and behaviors (e.g., Gini et al., 2007; Pozzoli et al., 2012), our findings highlight sex differences in bystanders' responses to bias‐based bullying: girls had higher behavioral intentions to help, lower masculinity/femininity threat, lower social contagion, and higher empathic responses. Future research is needed to better understand the societal and cognitive drivers of this difference. Overall, our findings extend previous research by identifying specific conditions (female, young age group) that the interventions are likely to have a positive impact. Thus, imagined and extended contact intervention methods seem to be more appropriate for younger females. For this particular intergroup context, older adolescents, and males, more rigorous interventions may be necessary, including, for instance, information regarding ingroup normative support (e.g., providing information about the number of ingroup members—heterosexual—who have outgroup—LGB—friends‐descriptive norms—and the normative support for cross‐group friendship‐injunctive norms; Gómez et al., 2018).

## 11 LIMITATIONS AND FUTURE DIRECTIONS

The current study has some limitations. First, the sample size may have affected the significance of our interaction effects, particularly the small number of male participants per cell. This means the findings regarding differences between male and female participants are underpowered and should be interpreted with caution. Thus, future studies could increase the sample size to guarantee sufficient power to test the expected effects. A strength of the current research is the focus on mediators specific to this intergroup context (i.e., social contagion, masculinity/femininity threat). However, they did not mediate the effects of the condition on bystanders' behavioral intentions. The contact literature tends to focus on examining more general mediators that are thought to be consistent across different intergroup contexts (e.g., intergroup anxiety, trust). Further research could explore these more general mediators in the context of homophobic bullyings, such as intergroup anxiety, which can potentially decrease after imagined and extended contact (e.g., Turner et al., 2007).

Also, in our studies, there were no observations of actual behavior. Rather, behavioral intentions were assessed in all studies. Previous research has revealed that intentions are important predictors of actual behaviors (e.g., Smith & McSweeney, 2007), still, future research must include measures of actual bystander behavior, to examine bystander responses more fully and accurately.

In the imagined contact study, participants found the scenario neutral and not openly positive, as we expected. Thus, future work should specifically incorporate the positive tone of the interaction in the instructions, since it is one of the key elements of effective imagined contact interventions (Crisp et al., 2009). In addition, in both studies, the majority of participants were revealed to have direct contact with outgroup members (i.e., gay/lesbian students), and past research suggests that children with higher levels of direct contact may not benefit from extended contact interventions (Cameron et al., 2011). Thus, extended contact interventions may be more effective in less heterogeneous schools, where adolescents are less exposed to sexual orientation diversity.

Moreover, sex‐matched vignettes of name‐calling homophobic bullying were presented to participants. We argue that it would be useful for future research to consider gender effects on bystander responses, while including, the aggressor, victim, and bystanders' gender to examine how bystander intentions may vary depending on the gender of the aggressor, victim, and bystander. In schools, although peers might group together by gender, it is possible that bullying could occur across gender, with both male and female bystanders present. Previous research conducted with young people (undergraduates) has examined this question and found the number of bystanders and their gender identity influence helping interventions (Levine & Crowther, 2008). Specifically, increasing the group size of bystanders, encouraged female intervention to help female victims, when the other bystanders were women and not when they were men. Conversely, increasing the number of male bystanders did not produce greater bystander intervention to help male victims. However, when the victim was female, male bystanders were more likely to intervene when more women were present (Levine & Crowther, 2008). As we observed in the current work, different intergroup processes and interventions may work differently and with different levels of effectiveness based on gender identity. Further research is required to account for these potential gender differences to develop more effective interventions, suitable for both female and male adolescents. Moreover, although some literature in empathy indicates that children and youth (especially females) show more affective empathy for same‐sex others than for other‐sex others (e.g., Stuijfzand et al., 2016), we acknowledge that it would be interesting to investigate different‐sex empathic responses.

Finally, and despite these limitations, our findings have potential implications for antibullying interventions. This research highlights the importance of the developmental intergroup context in bystanders' responses to bullying episodes, stressing the importance of developing and implementing appropriate anti‐bullying interventions in school‐based interventions that embrace sexual minority adolescents. The developmental trends and sex differences also illustrate how adolescents vary in their behavioral intentions as bystanders, with boys having fewer intentions to help than girls, and also having more social contagion concerns and more threat to masculinity. Males and older adolescents could benefit from more tailored anti‐bullying programs focused on homophobic bullying and masculine norms and behaviors, to promote more assertive bystanders among all students who witness these aggressive behaviors. Importantly, imagined and extended contact can be promising tools for school‐based interventions to promote more assertive and empathic bystanders in the school context, particularly with younger female adolescents.

## CONFLICT OF INTEREST

The authors declare no conflict of interest.

## Supporting information

Supporting information.

## Data Availability

The data that support the findings of this study are available on request from the corresponding author. The data are not publicly available due to privacy or ethical restrictions.

## ENDNOTES

^1^
For exploratory reasons, we assessed participants' quantity of direct contact (i.e., “How many gay/lesbian friends do you have”; on a 5‐point scale; adapted from Binder et al., 2009). Most of the participants reported having direct contact with the outgroup (i.e., Experiment 1: 53% have 1–3 outgroup friends; Experiment 2: 45.4% have 1–3 outgroup friends).

^2^
See Supporting Information for full measures.
