# Supplementary material for: Imagined and extended contact experiences and adolescent bystanders' behavioral intentions in homophobic bullying episodes
Source: Aggress Behav. 2022 Nov 4;49(2):110–26. doi: 10.1002/ab.22059 (PMC10099952; doi:10.1002/ab.22059)
Supplement: Supplementary file 1 — Supporting information. [file AB-49-110-s001.docx]

**Supporting Information**

Imagined and Extended Contact Experiences and Adolescent Bystanders’ Behavioral Intentions in Homophobic Bullying Episodes

**Supporting Information A**

**Vignette of a name-calling homophobic bullying episode.** Example of the vignette of a name-calling homophobic bullying episode for female participants: “Imagine that it is the end of the school day, you are walking down the corridor and you hear a student (Ana) shout a rude word against another student (Paula) because she is lesbian or because Ana thinks Paula is lesbian. What would you do?”. After the vignette, participants answered to the measures of empathic concern and assertive behavioral intentions.

**Empathic concern measure.** For female participants: “I feel sensitized by what happened to the bullied girl”, “I feel sorry for the bullied girl”, “I feel sympathy for the bullied girl”, “I feel moved by what happened to the bullied girl”.

For male participants: “I feel sensitized by what happened to the bullied boy”, “I feel sorry for the bullied boy”, “I feel sympathy for the bullied boy”, “I feel moved by what happened to the bullied boy”.

**Social contagion concerns** **Measure.** For female participants: “If I was hanging out with a lesbian person, I would worry that other people would think I was lesbian, too”; “I was worried that others would think I was lesbian if they knew I was friends with a lesbian person”; “It would bothered me if other people mistakenly thought I was lesbian”; “If I was going out (e.g., cinema) with a lesbian person, I would worry that people would think we were on a date”; “If I had to interact with a lesbian girl, I was worried she would flirt with me.”; “If I was friendly with a lesbian person, she would likely mistake my friendliness for dating/"flirtation"”; “If I were to become friends with a lesbian person, I would be concerned that she might think I was lesbian too.”; “If I was studying next to a lesbian person, I would want her to know that I am straight.”.

For male participants: “If I was hanging out with a gay person, I would worry that other people would think I was gay, too”; “I was worried that others would think I was gay if they knew I was friends with a gay person”; “It would bothered me if other people mistakenly thought I was gay”; “If I was going out (e.g., cinema) with a gay person, I would worry that people would think we were on a date”; “If I had to interact with a gay boy, I was worried he would flirt with me.”; “If I was friendly with a gay person, he would likely mistake my friendliness for dating/"flirtation"”; “If I were to become friends with a lesbian person, I would be concerned that he might think I was gay too.”; “If I was studying next to a gay person, I would want him to know that I am straight.”.

**Masculinity/femininity threat measure.** For female participants: “I would feel my femininity threatened if a lesbian girl flirted with me”; “If a lesbian girl hit on me, I would be disgusted”; “A girl must defend herself when a lesbian girl wants to hook up with her”.

For male participants: “I would feel my masculinity threatened if a gay boy flirted with me”; “If a gay boy hit on me, I would be disgusted”; “A boy must defend himself when a gay boy wants to hook up with him”.

**Supporting Information B**

**Imagined Contact Instructions (Experiment 1)**

Participants in the imagined contact condition were asked to imagine: ‘Please spend the next five minutes imagining that you are talking to a gay boy/lesbian girl [sex matched to participant] who sat next to you on the train. You spend about thirty minutes chatting until you reach your stop and leave the train. During the conversation you find out some interesting and unexpected things about him/her’. Participants were then instructed to ‘List the interesting and unexpected things you discovered about him/her following the conversation you just imagined’. Participants assigned to the control condition were asked: ‘Please spend the next five minutes imagining that you are on a three-day hiking trip in the south of Portugal. During the trip you arrive unexpectedly at a secluded bay’. Participants were then instructed to ‘List the different things that you saw in the scene you just imagined’.

**Supporting Information C**

**Extended Contact Instructions (Experiment 2)**

Extended contact was manipulated through fabricated entries on an Internet forum in which an ingroup member (i.e., heterosexual) described his/her positive, negative or absence of contact with a member of the outgroup (i.e., homosexual). All participants were presented with an excerpt that started with an entry posted by a supposed lesbian or gay student who was moving to the school and asked the online community about the school environment since she/he had problems in her/his current school related to her/his sexual orientation. After that, all participants read one of three different replies to this message provided by a heterosexual student from their school, depending on the valence of extended contact condition and participants sex. For example, female participants in negative extended contact condition read: “(…) The school environment is so so… sometimes we have some issues. For example, my boyfriend has two friends, who are girlfriends. They talk a lot and they always want to be the center of attention and I don’t like it at all”. Female participants assigned to the positive extended contact condition read: “(…) The school environment is peaceful. People are laid-back and we get along well. For example, my boyfriend has two friends, who are girlfriends and they never had any problems at school. They are really cool and we usually go to the cinema together and with other people…”. Female participants in control condition read: “(…) The school environment is peaceful, people are laid-back and we get along well, but I don’t know if there are such problems…”.

**Supporting Information D**

**Indexes of moderated mediation (Experiment 1):** experimental condition as the predictor, age as moderator, social contagion concerns as mediator: -0.00, 95% CI [-0.08, 0.06], threat as mediator: -0.00, 95% CI [-0.07, 0.07], empathic concern as mediator: -0.05, 95% CI [-0.33, 0.20]. Experimental condition as the predictor sex as moderator, social contagion concerns as mediator: -0.01, 95% CI [-0.10, 0.05], threat as mediator: -0.00, 95% CI [-0.08, 0.05], empathic concern as mediator: 0.04, 95% CI [-0.28, 0.29].

**Indexes of moderated mediation (Experiment 2):** positive extended contact as the predictor, age as moderator, social contagion concerns as mediator: -0.05, 95% CI [-0.18, 0.07], threat as mediator: -0.08, 95% CI [-0.24, 0.03], empathic concern as mediator: -0.12, 95% CI [-0.36, 0.12]. Positive extended contact as the predictor sex as moderator, social contagion concerns as mediator: 0.05, 95% CI [-0.06, 0.19], threat as mediator: 0.03, 95% CI [-0.06, 0.15], empathic concern as mediator: 0.01, 95% CI [-0.23, 0.25]. Negative extended contact as the predictor and age as moderator, social contagion concerns as mediator: 0.02, 95% CI [-0.12, 0.15], threat as mediator: 0.05, 95% CI [-0.04, 0.18], empathic concern as mediator: 0.12, 95% CI [-0.12, 0.37]. Negative extended contact as the predictor and sex as moderator, social contagion concerns as mediator: 0.05, 95% CI [-0.06, 0.20], threat as moderator: 0.03, 95% CI [-0.06, 0.15], empathic concern as mediator: 0.01, 95% CI [-0.22, 0.25].
